# PhyloGeoTool: interactively exploring large phylogenies in an epidemiological context

**DOI:** 10.1093/bioinformatics/btx535

**Published:** 2017-08-26

**Authors:** Pieter Libin, Ewout Vanden Eynden, Francesca Incardona, Ann Nowé, Antonia Bezenchek, EucoHIV Study Group, Anders Sönnerborg, Anne-Mieke Vandamme, Kristof Theys, Guy Baele

**Affiliations:** 1 Department of Microbiology and Immunology, Rega Institute for Medical Research, Clinical and Epidemiological Virology, KU Leuven – University of Leuven, Leuven, Belgium; 2 Department of Computer Science, Vrije Universiteit Brussel, Brussels, Belgium; 3 EuResist Network, Rome, Italy; 4 InformaPRO, Rome, Italy; 5 www.eucohiv.org; 6 Karolinska Institute, Karolinska University Hospital, Stockholm, Sweden; 7 Center for Global Health and Tropical Medicine, Unidade de Microbiologia, Instituto de Higiene e Medicina Tropical, Universidade Nova de Lisboa, Lisbon, Portugal

## Abstract

**Motivation:**

Clinicians, health officials and researchers are interested in the epidemic spread of pathogens in both space and time to support the optimization of intervention measures and public health policies. Large sequence databases of virus sequences provide an interesting opportunity to study this spread through phylogenetic analysis. To infer knowledge from large phylogenetic trees, potentially encompassing tens of thousands of virus strains, an efficient method for data exploration is required. The clades that are visited during this exploration should be annotated with strain characteristics (e.g. transmission risk group, tropism, drug resistance profile) and their geographic context.

**Results:**

PhyloGeoTool implements a visual method to explore large phylogenetic trees and to depict characteristics of strains and clades, including their geographic context, in an interactive way. PhyloGeoTool also provides the possibility to position new virus strains relative to the existing phylogenetic tree, allowing users to gain insight in the placement of such new strains without the need to perform a de novo reconstruction of the phylogeny.

**Availability and implementation:**

https://github.com/rega-cev/phylogeotool (Freely available: open source software project).

**Supplementary information:**

Supplementary data are available at *Bioinformatics* online.

## 1 Introduction

Expanding and intensifying sequencing efforts for the management of infectious diseases along with the generation of large-scale databases of clinical and demographical information provide unprecedented opportunities for the surveillance of epidemics and outbreaks of viral pathogens. Mapping the origin and dynamics of epidemics in space and time is becoming feasible as geo-tagged and time-stamped sequence data are now part of routine clinical care. Tracking the geographical spread and the relationship to specified characteristics for distinct virus clades (e.g. transmission risk group, tropism, drug resistance profile) can help to improve our understanding of such outbreaks. Computational and methodological advances now allow to infer phylogenies of tens of thousands of sequences (Liu *et al.*, 2011) and applications have been developed to visualize such large phylogenetic trees (de Vienne, 2016; Huson and Scornavacca, 2012). However, efficient means to visually navigate through these large phylogenies and the annotated information (e.g. virus and patient data) are currently still lacking. Further, fast and accurate placement of novel virus sequences onto an existing phylogenetic tree can provide valuable insights for outbreak detection, by relating evolutionary dynamics to epidemiological and clinical characteristics.

## 2 Features

**Fig. 1:**
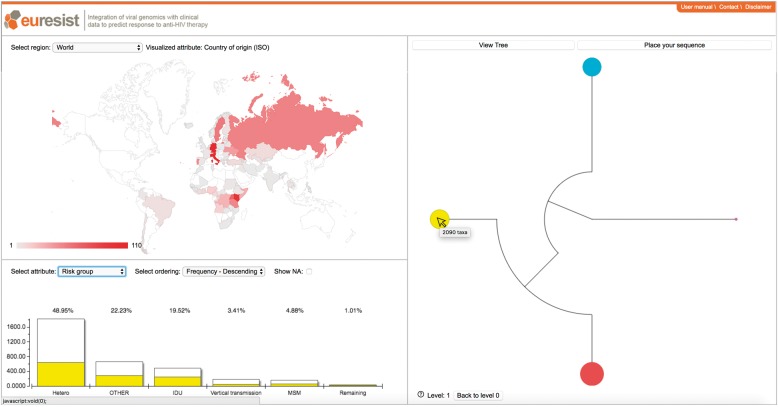
The PhyloGeoTool graphical user interface. The upper left panel shows the geographical distribution of the samples present in the selected cluster. The lower left panel shows the distribution for a selected trait of interest; white bars show the distribution for the entire dataset for that level of the tree, whereas the colored bars show the distribution for a specific selected cluster and are annotated by their respective percentage. The right panel shows the clustered phylogenetic tree and allows to perform phylogenetic placement

We present PhyloGeoTool, an application to interactively navigate large phylogenies and to explore associated clinical and epidemiological data. PhyloGeoTool implements an algorithm that automatically partitions a phylogeny into an optimal number of clusters, thereby recursively partitioning each identified cluster (see Section 3). A graphical user interface provides a concise visualization of the initial tree of clusters. Subsequent levels of the phylogeny are visualized upon the selection of a specific cluster (Fig. 1), with an option to show their respective positions within the entire phylogeny (not shown). At each partitioning level of the phylogenetic tree, an overview of sequence attributes is provided. A map shows the geographic distribution of sampling and a bar chart shows the distribution of the attribute that was selected by the user. In addition, hovering over a particular cluster activates a bar chart which presents attribute information for that cluster in relation to the rest of the clusters (Fig. 1).

PhyloGeoTool only requires the user to provide a phylogenetic tree and attribute information for each taxon, without the need for an underlying database structure. Given that processing large phylogenies is a time-consuming task, a phylogeny is partitioned prior to the deployment of the web application. This enables the exploration of the phylogenetic tree to be instantaneous for the user. Further, the inclusion of novel sequence data does not require the re-estimation of the phylogeny or partitioning, as PhyloGeoTool supports the fast and accurate phylogenetic placement of submitted virus sequences in the existing phylogeny using pplacer (Matsen *et al.*, 2010) (section ‘Phylogenetic placement’ in Supplementary Material). PhyloGeoTool is implemented as a web application to offload the installation and computational burden to the hosting server.

## 3 Materials and methods

We here present an algorithm that partitions the binary phylogenetic tree into clusters using a recursive approach. Combining such an approach to identify clusters of sequences with a progressive zooming approach ensures an efficient and interactive visual navigation of the entire phylogenetic tree. To partition a binary tree T into *k* clusters, the following algorithm was devised. Intuitively, the binary tree is partioned recursively using the cluster sizes as clustering criterium. Starting at the root of the tree T, the first cluster consists of its left child and all its descendants (i.e. the ‘left’ part of T), while the second cluster consists of its right child and all its descendants (i.e. the ‘right’ part of T). These clusters are added to a set C, that is ordered by descending cluster size (i.e. the number of tree leaves that each cluster covers). The largest cluster from C is removed and its corresponding tree is split at the root, creating two new clusters corresponding to the resulting subtrees. These two new clusters are subsequently added to C. This process is repeated until the maximum number of clusters is reached (i.e. |C|=k).

While this method results in the partitioning of T in *k* clusters, a value for *k* that ensures the presence of well-defined clusters still needs to be determined. The subtype diversity ratio (SDR) provides a measure to score a particular clustering C of T and is defined as the ratio of the mean intra-cluster pairwise distance to the mean inter-cluster pairwise distance (Rambaut *et al.*, 2001). Therefore, low intra-pairwise distances relative to inter-pairwise distances imply the presence of well-defined clusters (Archer and Robertson, 2007). To determine the optimal value for *k*, the SDR(T,k) function is analyzed from *k* = 2 (i.e. the minimal cluster) to *k* = 50 (i.e. the maximal cluster size). In this process, two cases are discerned: the SDR function exhibits a descending trend over the entire domain or a clear local minimum can be found when analysing the SDR function. In the first case, *k* is found optimal where the loss in SDR is maximal: such a *k* can be found by considering all SDR scores for $k\to k_{max}$ (i.e. *k_max_* = 50, the maximal cluster size) and selecting *k* where the curvature of SDR(T,k) is maximal (Equation 1). In the second case, the local SDR minimum is selected. We refer to the Supplementary Material (section ‘SDR function analysis’) for more details on the SDR function analysis and some examples that demonstrate the process.

(1) $$k_{optimal}=\underset{k}{\text{argmax}}[SDR\prime \prime (T,k)].$$

## 4 Application and future perspectives

To illustrate this, we have evaluated PhyloGeoTool in the context of HIV-1 using data available within the EuResist Integrated Data Base (Fig. 1) (Zazzi *et al.*, 2012). This database contains virus genotypes, clinical responses and epidemiological markers of more than 66.000 patients from 12 different countries. A public version of the web application operating on the EuResist dataset is available at http://phylogeotool.gbiomed.kuleuven.be/euresist/.

To demonstrate PhyloGeoTool‘s potential, we present a case study concerning transmitted HIV-1 drug resistance in Europe using the EuResist PhyloGeoTool instance (details in the ’Case study’ section of the Supplementary Material). We investigate the prevalence of transmitted drug resistance (RegaDB software; Libin *et al.*, 2013) and its association with geography, HIV-1 subtype (Rega HIV subtyping tool; Alcantara *et al.*, 2009) and particular clades in the phylogenetic tree. As we report in Supplementary Material, observed trends were in agreement with a recent European study concerning transmitted drug resistance (Hofstra *et al.*, 2016).

In addition, we have evaluated PhyloGeoTool in the context of Dengue virus (DENV). We have downloaded a dataset of 8125 envelope gene sequences covering all four DENV serotypes from Genbank and their attributes (i.e. serotype, genotype, sample source, country of origin and collection date). A public version of the web application operating on the Dengue dataset is available at http://phylogeotool.gbiomed.kuleuven.be/dengue/.

The evaluations, where our clustering algorithm was able to extract the expected clusters, show that PhyloGeoTool has the potential to act as an important tool to inform public health by providing support to visualize, navigate and study large sequence databases of viral pathogen with annotated data.

## Supplementary Material

Supplementary Data
