# Supplementary material for: PhyloGeoTool: interactively exploring large phylogenies in an epidemiological context
Source: Bioinformatics. 2017 Aug 26;33(24):3993–5. doi: 10.1093/bioinformatics/btx535 (PMC5860094; doi:10.1093/bioinformatics/btx535)
Supplement: Supplementary Data [file btx535_supplement.pdf]

Supplementary information

This file provides supplementary information that accompanies the manuscript ‘PhyloGeoTool: interactively exploring large phylogenies in an epidemiological context’.

1. SDR function analysis 2
    1.1. Example data sets 2
2. Phylogenetic placement 8
3. Case study 8
    3.1. High-level difference per subtype 8
    3.2. High-level difference within a single subtype 12
4. EucoHIV Study group 16
5. References 19

1. SDR function analysis

In the main manuscript, we describe an algorithm to partition a phylogeny into *k* clusters. In order to find an optimal clustering of the phylogeny, a value of *k* that ensures the presence of well-defined clusters hence needs to be determined. To this end, we use the subtype diversity ratio (SDR), which provides a statistic to score a particular clustering and is defined as the ratio of the mean intra-cluster pairwise distance to the mean inter-cluster pairwise distance (Rambaut et al., 2001).

To determine the optimal value for *k*, the SDR function is analysed from *k*=2 (i.e. the minimal cluster size) to *k*=50 (i.e. the maximal cluster size). In this process, two cases are discerned: the SDR function either exhibits a descending trend over the entire domain or a clear local minimum can be found when analysing the SDR function. To discern between the two cases, the first derivative of the SDR values is considered. When less than 20% of the SDR derivatives are positive, the SDR trend is considered to be descending.

In the first case, *k* is found optimal where the loss in SDR is maximal: such a *k* can be found by considering all SDR scores and selecting *k* where the curvature of the SDR function is maximal. In the second case, the first local SDR minimum is simply selected.

1.1. Example data sets

We first present an example application of our SDR function analyses based on a large Italian clade from the EuResist database (Zazzi et al., 2012). In Figure S1, we show the computed SDR function from *k*=2 to *k*=50. In Figure S2, we show the first derivatives of the SDR function. These first derivatives allow us to conclude that the SDR function exhibits a downward trend. As the SDR function exhibits a downward trend, we need to analyze its curvature. We do this by analyzing the second derivatives of the SDR function, as shown in Figure S3.

We go on to present a second example from the EuResist database (Zazzi et al., 2012), this time using the entire database. In Figure S4, we show the computed SDR function from *k*=2 to *k*=50. In Figure S5, we show the first derivatives of the SDR function. These first derivatives allow us to conclude that the SDR function does not exhibit a downward trend. Therefore, we find the optimal number of clusters at the first local minimum of the SDR function.

Figure S1. Example of an SDR function that exhibits a descending trend, as inferred on a large Italian clade from the EuResist database.

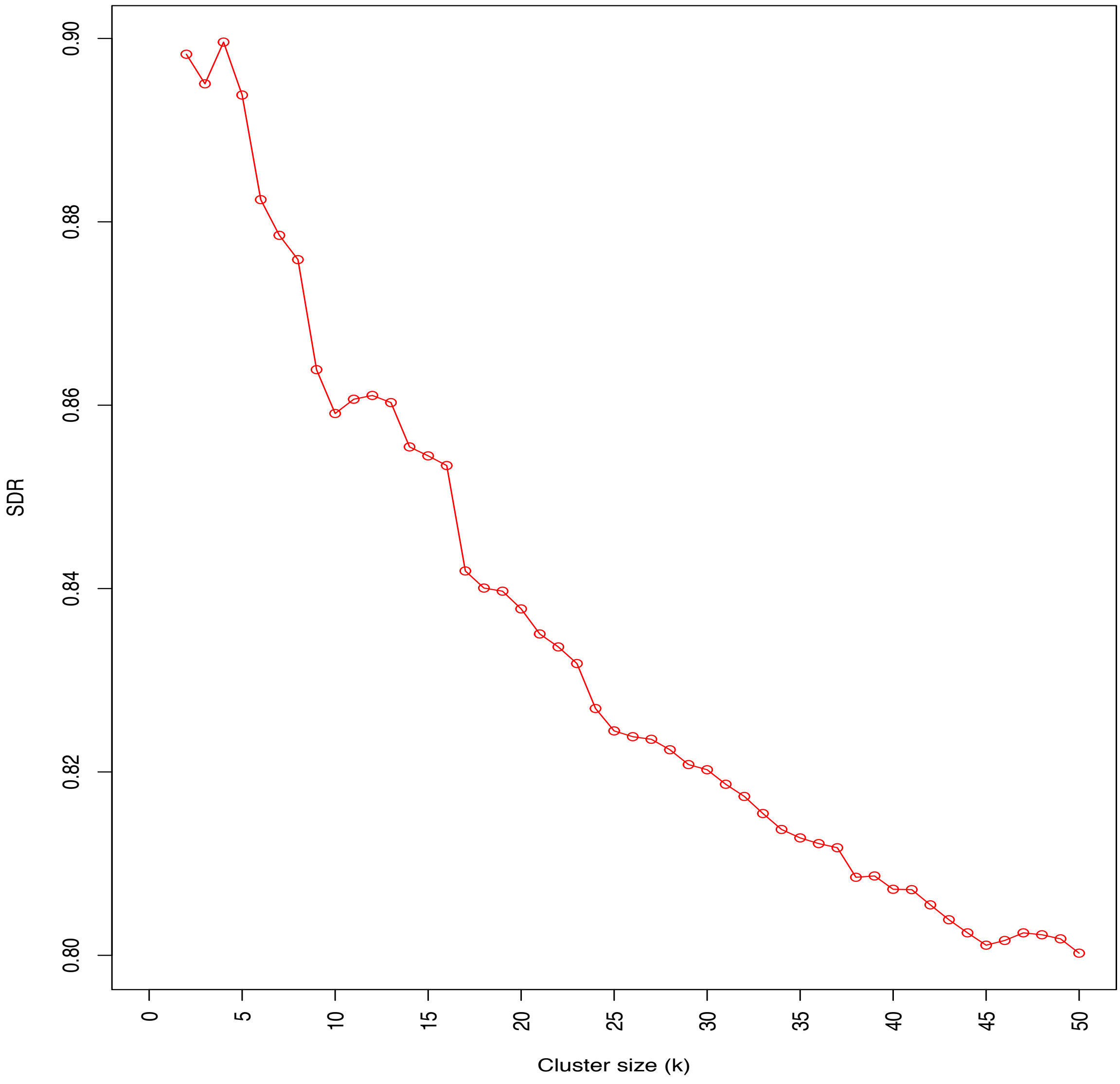

Figure S2. The first derivatives of the considered SDR values of the Italian clade are shown. Less than 20% of the derivatives are positive, leading us to conclude that the SDR curve exhibits a descending trend. Therefore, to determine the optimal number of clusters, we need to perform an analysis of the SDR curve's curvature: this analysis is visualized and explained in Figure S3.

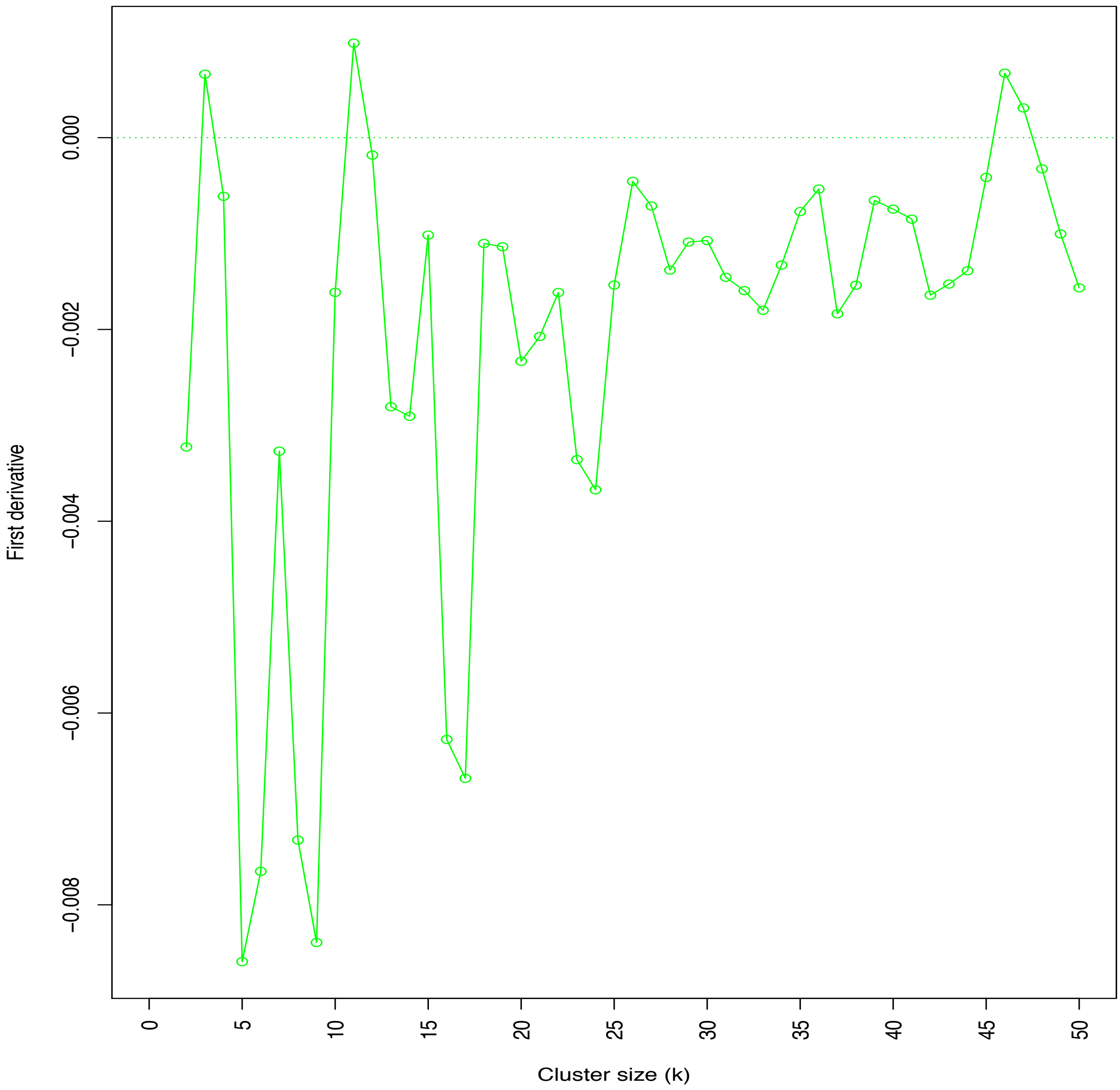

Figure S3. The SDR curvature is analysed by considering the second derivate of the SDR function. The loss in SDR is maximal where the second derivate (SDR'') is maximal, in this figure the second derivative is maximal at *k*=17, as shown by a vertical dotted line. Note that SDR'' takes on the domain [3,50].

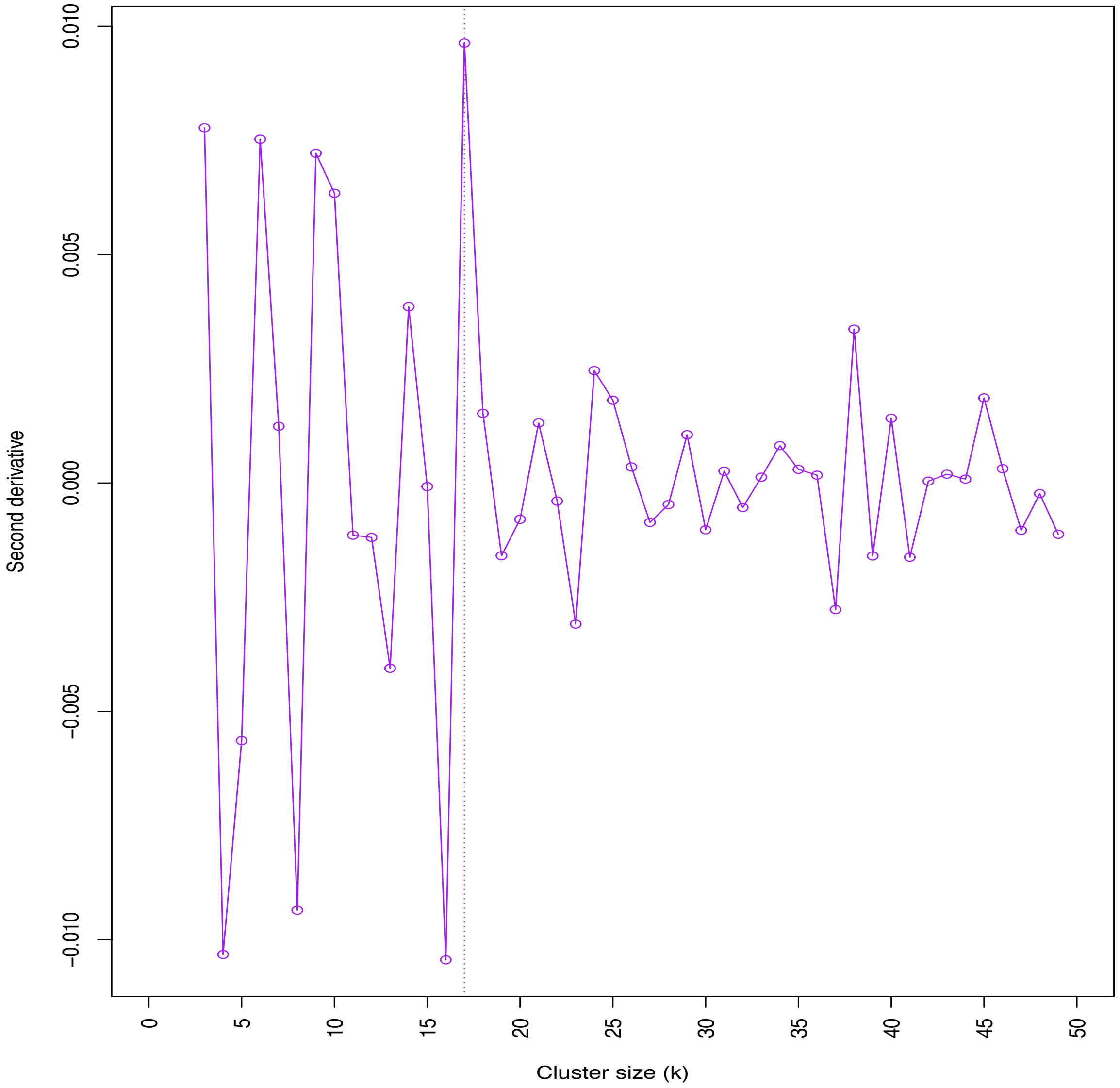

Figure S4. Example of an SDR function as inferred based on the entire EuResist phylogeny. A local minimum can be observed at *k*=3 on the SDR curve.

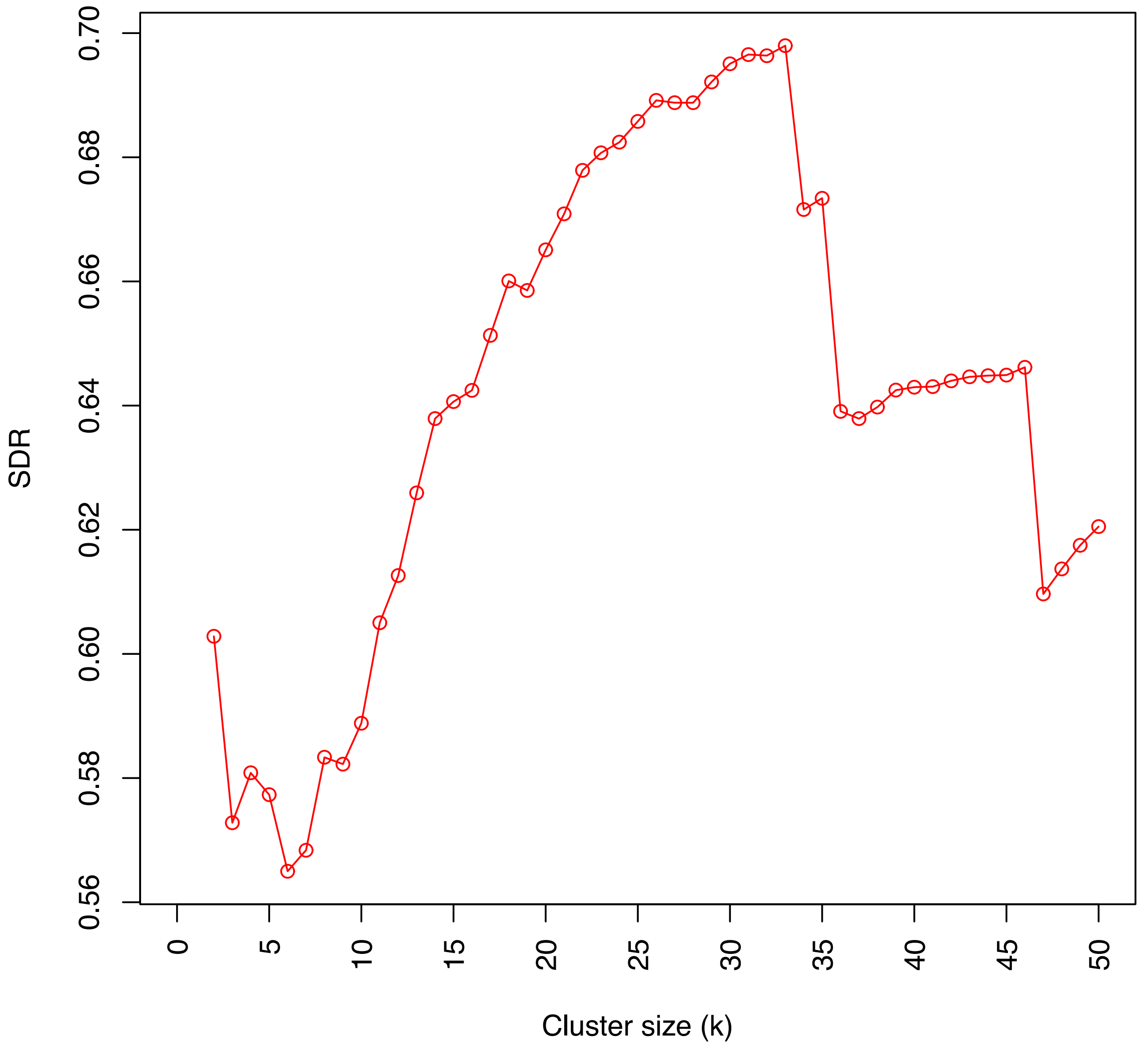

Figure S5. The first derivatives of the considered SDR values are shown. More than 20% of the derivatives are positive, leading us to conclude that the SDR curve does not exhibit a descending trend. Therefore, the number of clusters is optimal at the first local minimum, the SDR function in Figure S4 shows that this minimum can be found at *k=3*.

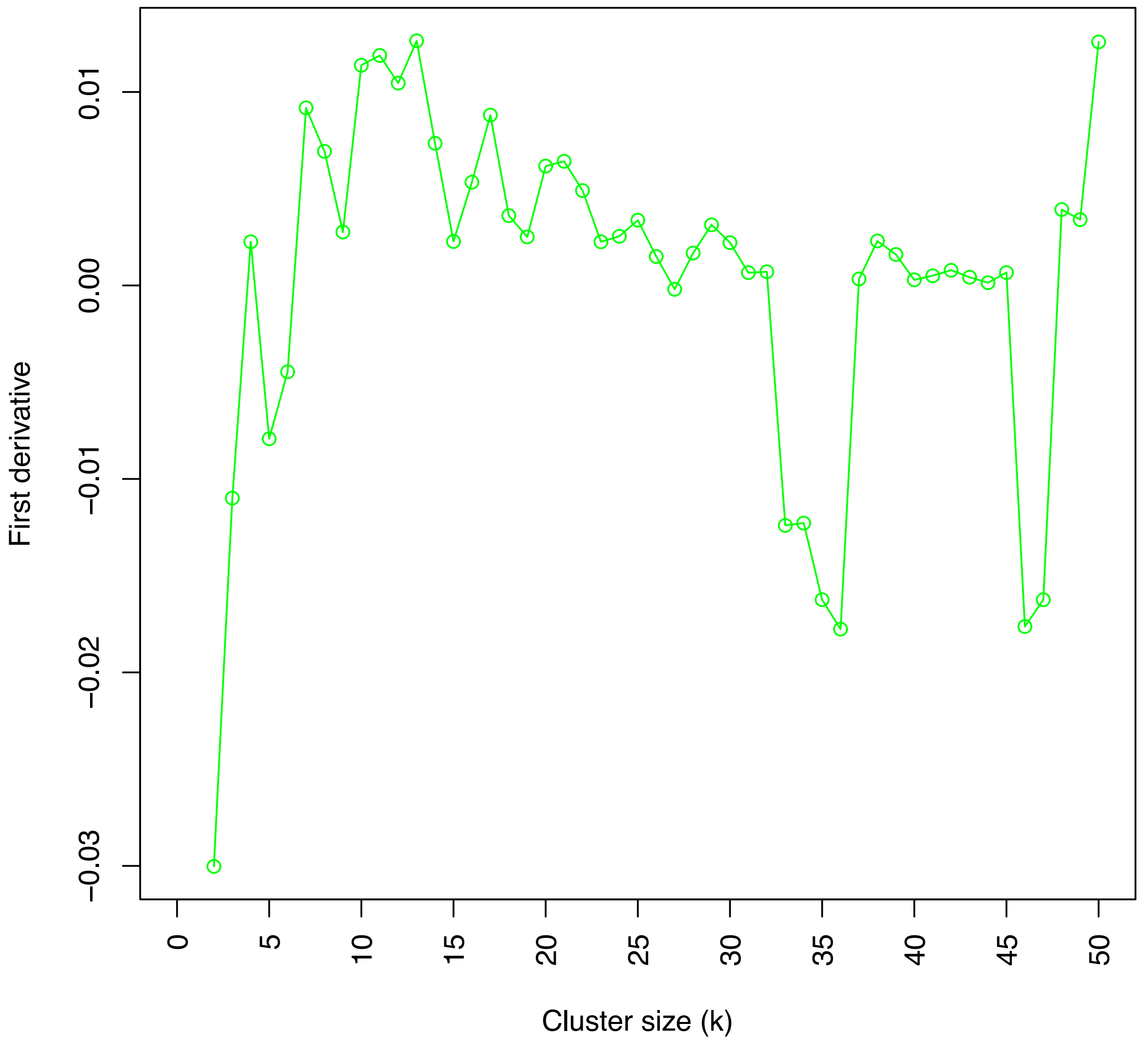

2. Phylogenetic placement

Phylogenetic placement can be used to determine the location of query sequences in a phylogenetic tree. pplacer is a popular program that implements phylogenetic placement on maximum-likelihood trees (Matsen et al., 2010). Fast and accurate placement of novel virus sequences into an existing phylogenetic context can provide valuable insights for outbreaks detection. Therefore, phylogenetic placement is implemented in the PhyloGeoTool to allow users to place their query sequences on the clustered phylogenetic tree.
To enable phylogenetic placement, a pplacer reference package is constructed. This package contains references to the used phylogenetic tree, a log transcript that was generated while the phylogenetic tree was inferred and a reference to the sequence alignment. This reference package allows pplacer to compute a confidence score for each leaf in the phylogenetic tree. Based on this set of scores, a new phylogenetic tree is generated where the query sequence is placed at the most likely location in the reference tree. Based on this newly constructed tree, the location in the clustered phylogeny is determined. This location is visualized onto the clustered tree in the PhyloGeoTool user interface.

3. Case study

To demonstrate PhyloGeoTool's potential, we present a case study concerning the transmission of HIV-1 drug resistance in Europe using the EuResist PhyloGeoTool instance.[1] In this case study, we investigate the prevalence of transmitted drug resistance (TDR) and its association with geography, HIV-1 subtype and particular clades in the phylogenetic tree.

In order to study transmitted drug resistance (TDR), we queried virus sequences from treatment-naive patients (i.e. patients that have not yet experienced therapy) from the EuResist database (Zazzi et al., 2010). For each treatment-naive patient, the presence of TDR was determined by checking for surveillance drug resistance mutations (SDRMs) as defined by the WHO (Bennett et al., 2008). TDR was scored 'True' when one or more SDRMs could be detected and otherwise scored 'False', using the RegaDB software (Libin et al., 2013). These TDR scores were added as an attribute to the EuResist PhyloGeoTool instance and subsequently analyzed using the PhyloGeoTool user interface. A value 'Treated' was assigned to sequences that were isolated during antiretroviral treatment. In the following subsections, we demonstrate that the PhyloGeoTool can be used to detect differences in TDR prevalence between HIV-1 subtypes and between clades within a single HIV-1 subtype.

3.1. High-level difference per subtype

The initial view of the EuResist PhyloGeoTool instance shows a clustering per subtype. Our clustering approach detects a large cluster of subtype B sequences (Figure S6, red cluster), a small cluster of subtype F (F1) sequences (Figure S6, light blue cluster), a medium-sized cluster of subtype G, A (A1) and C sequences (Figure S6, yellow cluster).

To illustrate PhyloGeoTool's use to study the surveillance of TDR, we compare the TDR score for nucleoside reverse transcriptase inhibitors (NRTI) between these clusters. This comparison reveals a large difference between the subtype B cluster and the other clusters, with the subtype B cluster exhibiting NRTI TDR of 12.3%, compared to a much lower NRTI

[1] http://phylogeotool.gbiomed.kuleuven.be/euresist/

TDR of 7.98% (yellow cluster) and 7.89% (light blue cluster) for the two other major clusters (Figure S7).

There is quite some heterogeneity in the NRTI TDR scores within the medium-sized cluster of subtype G, A (A1) and C sequences, which contains three nicely separated clusters grouped according to subtype (Figure S8): a cluster of subtype G sequences (Figure S8, red cluster), a cluster of subtype A (A1) sequences (Figure S8, yellow cluster), a cluster of subtype C sequences (Figure S8, light blue cluster) and a very small cluster of subtype H sequences (Figure S8, purple cluster).

Comparing the TDR score between these smaller clusters reveals additional differences between clusters (Figure S9): an NRTI TDR score of 10.05% for the subtype G cluster, an NRTI TDR score of 7.93% for the subtype A (A1) cluster, an NRTI TDR score of 5.34% for the subtype C cluster and an NRTI TDR score of 12.5% for the subtype H cluster (data not shown).

Note that these trends are in agreement with a recent European study concerning transmitted drug resistance (Hofstra *et al.*, 2016).

Note that these differences also illustrate the usefulness of PhyloGeoTool's phylogenetic placement method. This feature allows users to place their own sequences onto the existing phylogenetic tree. However, if certain traits of those sequences are unknown, they can be inferred through the properties of the cluster in which they end up being placed. Given that our EuResist instance of the PhyloGeoTool comes equipped with TDR scores for each cluster, phylogenetic placement of a sequence into one of the predetermined clusters allows users to infer a background about the expected level of TDR in that particular clade.

Figure S6. Top view (level: 0) of the EuResist PhyloGeoTool instance, showing a clustering per subtype as determined by our clustering approach. The red cluster contains almost exclusively subtype B sequences, while the light blue cluster contains only subtype F (F1) sequences and the yellow cluster contains subtype G, A (A1) and C sequences.

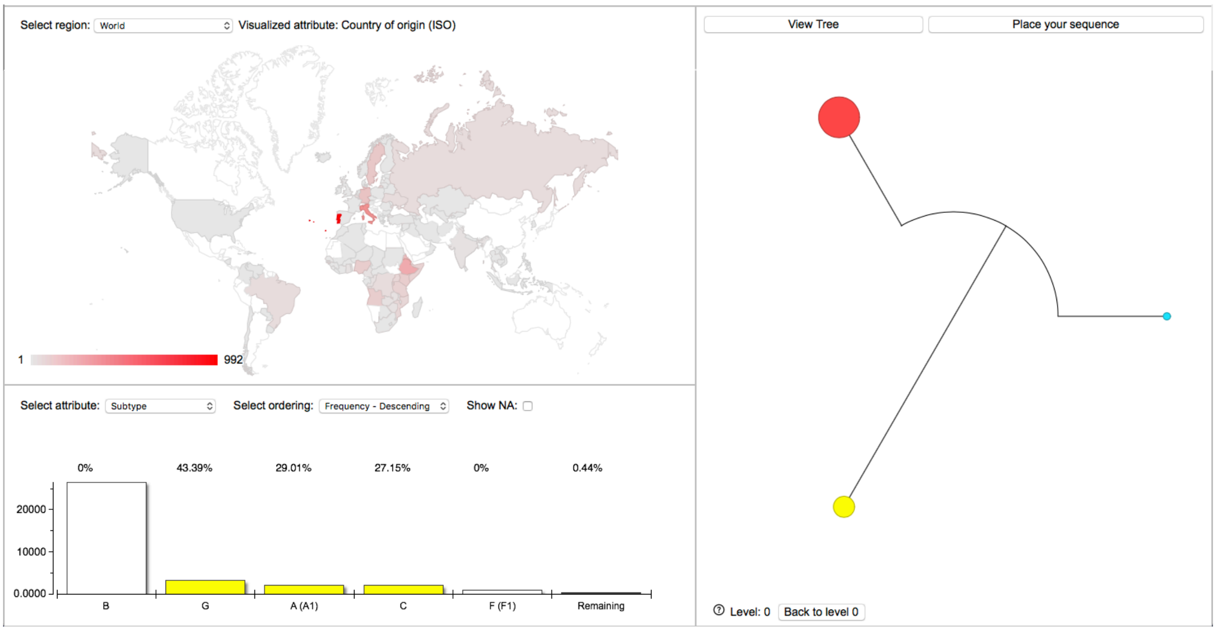

Figure S7. Composite figure showing the differences in TDR between different subtypes in the EuResist database. The top-level clustering (level: 0) shows a nucleoside reverse transcriptase inhibitor (NRTI) TDR of 12.3% for the subtype B cluster (in red), whereas the joint cluster of subtype G, A (A1) and C sequences (in yellow) shows an NRTI TDR of 7.98%. The light blue cluster shows an NRTI TDR of 7.89%.

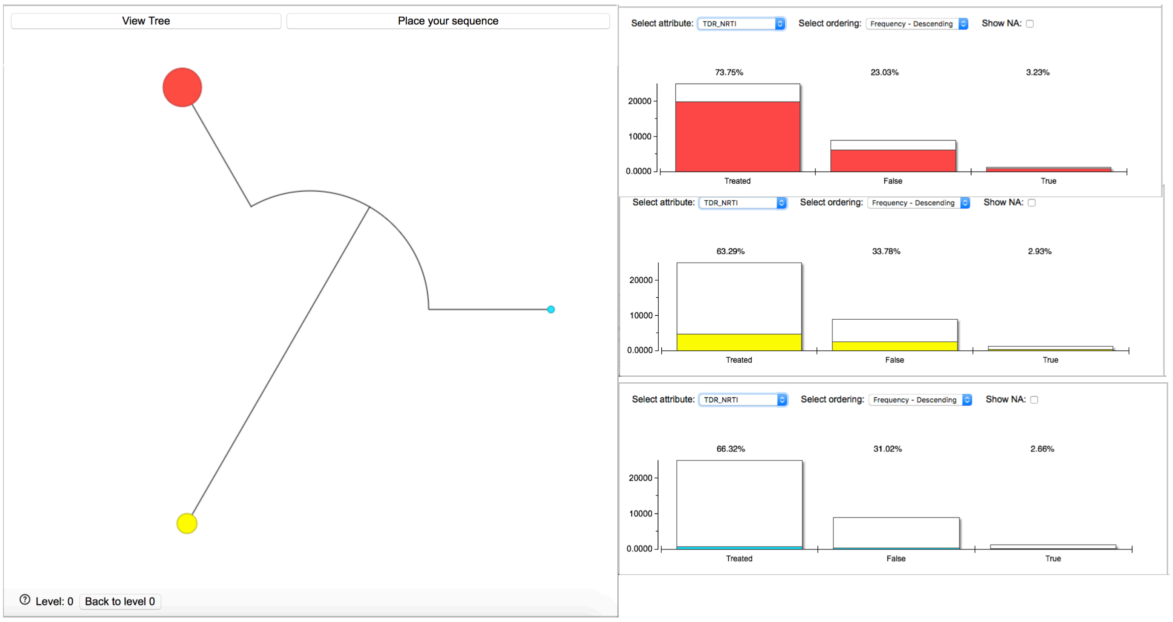

Figure S8. The cluster containing subtype G, A (A1) and C sequences (see Figure S7) itself contains three nicely separated clusters: subtype G (red), subtype A (A1) (yellow) and subtype C (light blue).

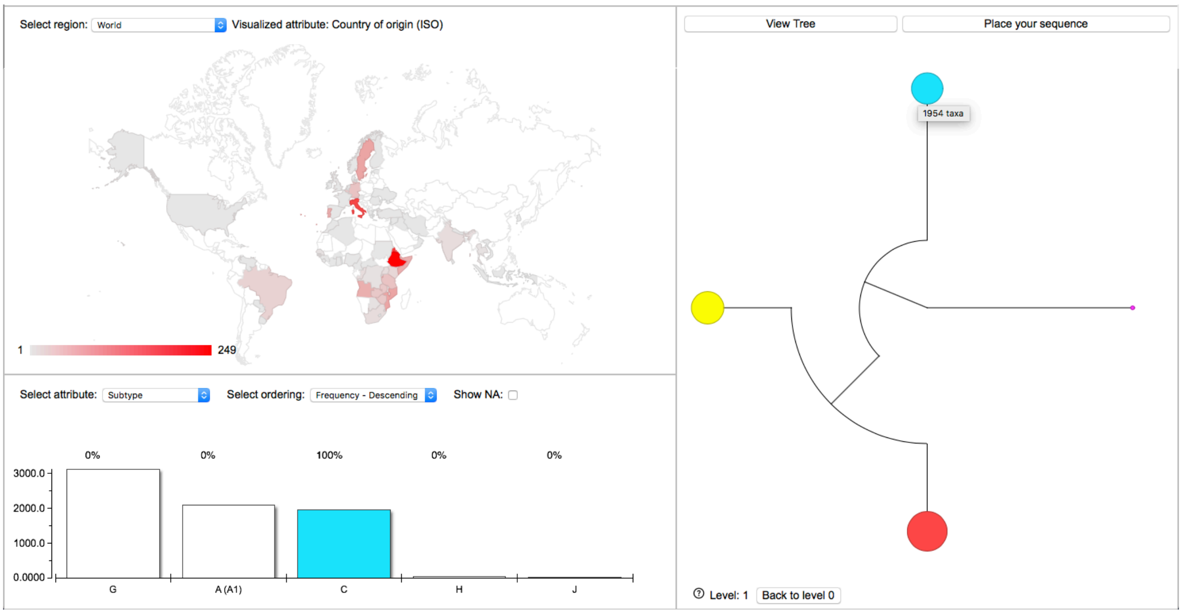

Figure S9. Composite figure, showing the differences in TDR between different subtypes in the EuResist database. The first-level clustering (level: 1) shows an NRTI TDR of 10.05% for the subtype G cluster (in red), an NRTI TDR of 7.93% for the subtype A (A1) cluster (in yellow) and an NRTI TDR of 5.34% for the subtype C cluster (in light blue).

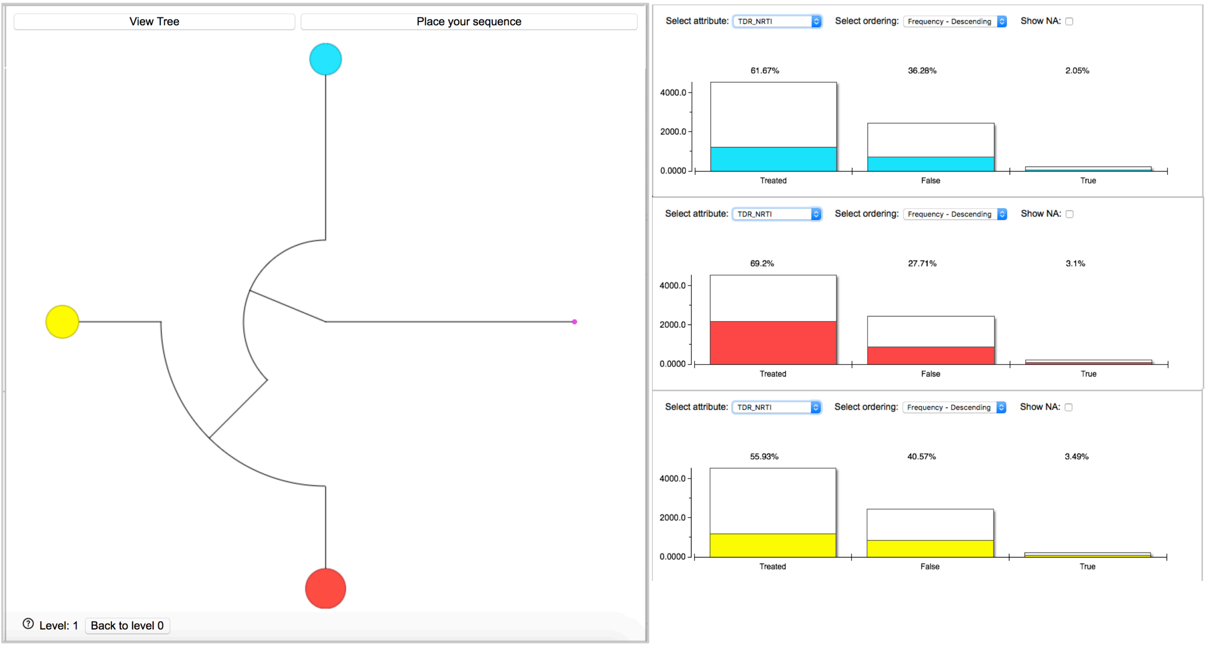

3.2. High-level difference within a single subtype

The example case shown above illustrates well how PhyloGeoTool can efficiently visualize differences between clades, such as HIV-1 subtypes, that are an intrinsic result of the clustering algorithm. Furthermore, variation in associated data can also been shown within a single HIV-1 subtype, in order to detect specific clades with high values of a characteristic of interest or to compare frequencies between groups (e.g. country, risk group) from where the samples were obtained.

To illustrate this feature, we have further explored the subtype B clade discussed above. We moved down the tree to level 5 (Figure S10) as a starting point. At this level, we observe that the patient population originates predominantly from Italy (65%), but also from Germany (12%), Portugal (5%) and Sweden (4%). Particularly interesting, is the yellow cluster, due to its heterogeneity in countries from which the patients originated, with Italy (49%), Germany (16%), Sweden (7.4%) and Portugal (6.9%) as main sources. The prevalence of NRTI TDR in treatment-naive patients for this cluster is 12%. In contrast, the red cluster consisted for more than 90% out of patients originating from Italy and had a NRTI TDR prevalence of 16%.

When we further descend into the yellow cluster, the clustering algorithm identified a range of clusters, heterogeneous in different characteristics (not shown). For the example here, we focused on the dark-green cluster, which contained 360 patients (Figure S11). Patients within this cluster primarily originated from Italy (37.8%) and Germany (38.2%), and displayed an overall NRTI TDR prevalence of 8% in treatment-naïve patients. Within this dark-green cluster, the clustering algorithm identified 6 distinct clusters (Figure S12). These clusters differ substantially with respect to country of origin and NRTI TDR prevalence (Figure S13). The patient population originating from Italy exhibits higher NRTI TDR levels than patients originating from Germany, as can be inferred from the characteristics of each clusters. The pink cluster had the highest prevalence of NRTI TDR (20.3%), in this cluster 77.5% of patients originate from Italy. The yellow cluster had a NRTI TDR prevalence of 12.8%, in this cluster 37% of patients originate from Germany while 33 % originate from Italy. The blue cluster had a NRTI TDR prevalence of 11.2%: in this cluster 35.2% of patients originate from Italy while 29.6% of patients originate from Sweden. Differently, the red and green clusters both had a NRTI TDR prevalence of 0%, and patients originated mainly from Germany (65% and 76% respectively).

Figure S10. A deeper exploration of the subtype B clade, ending up at level 5, through consecutive clicking of the cluster with the highest number of taxa. The left panel shows the presence of the identified clusters in the phylogenetic tree, while the right panel presents an overview of the different clusters.

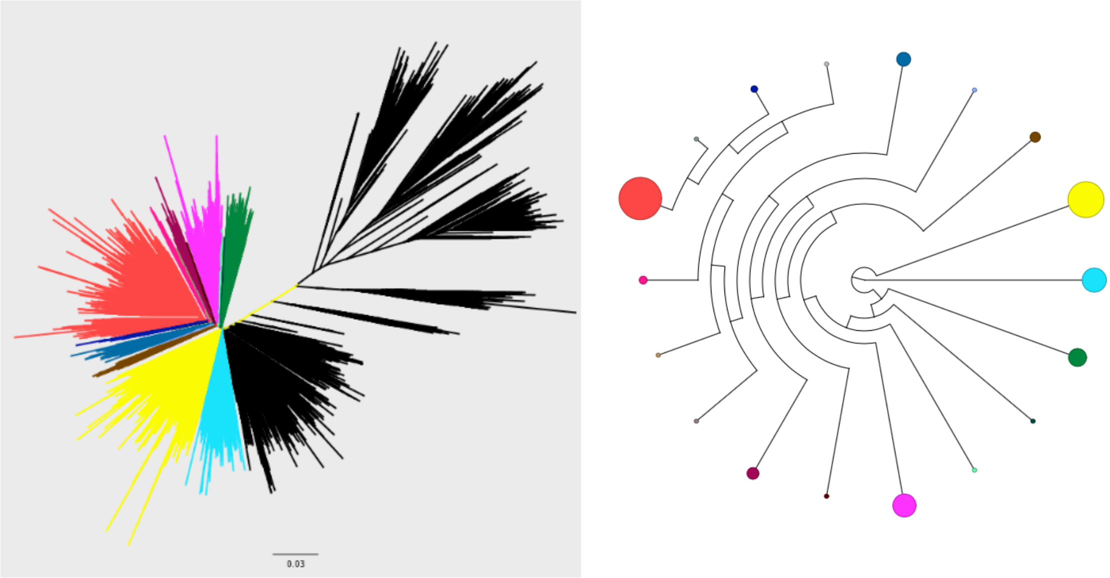

Figure S11. Overview of clusters at level 6, accessed by clicking on the yellow cluster at level 5. The dark-green cluster is of interest here.

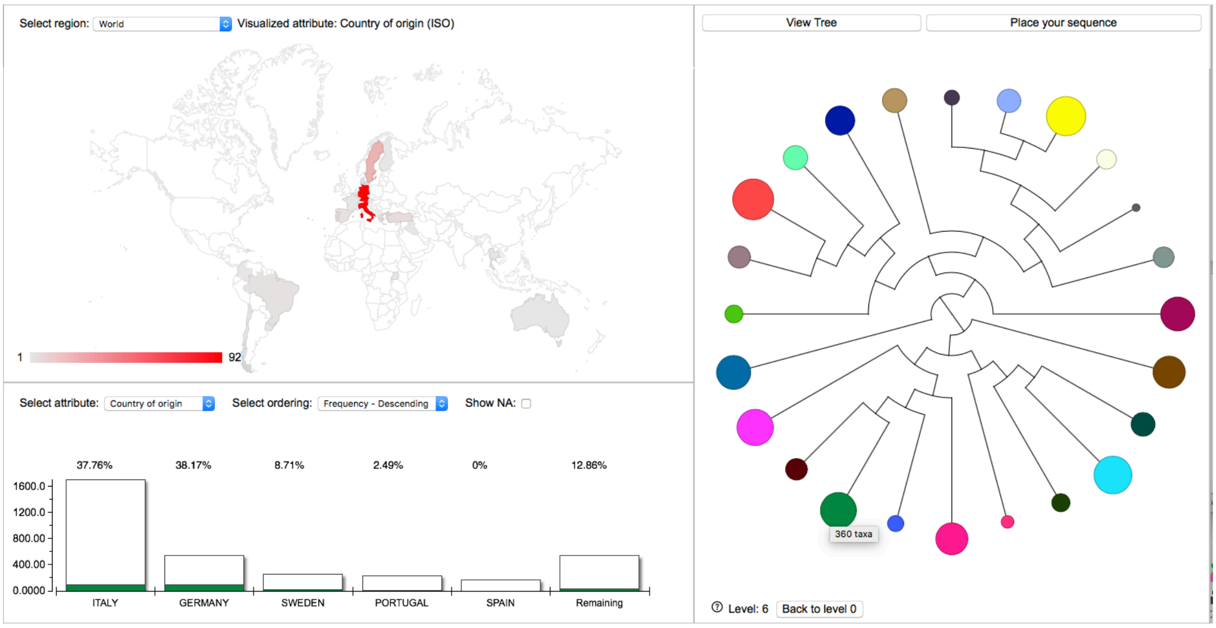

Figure S12. Overview of clusters at level 7, accessed by clicking on the yellow cluster at level 6.

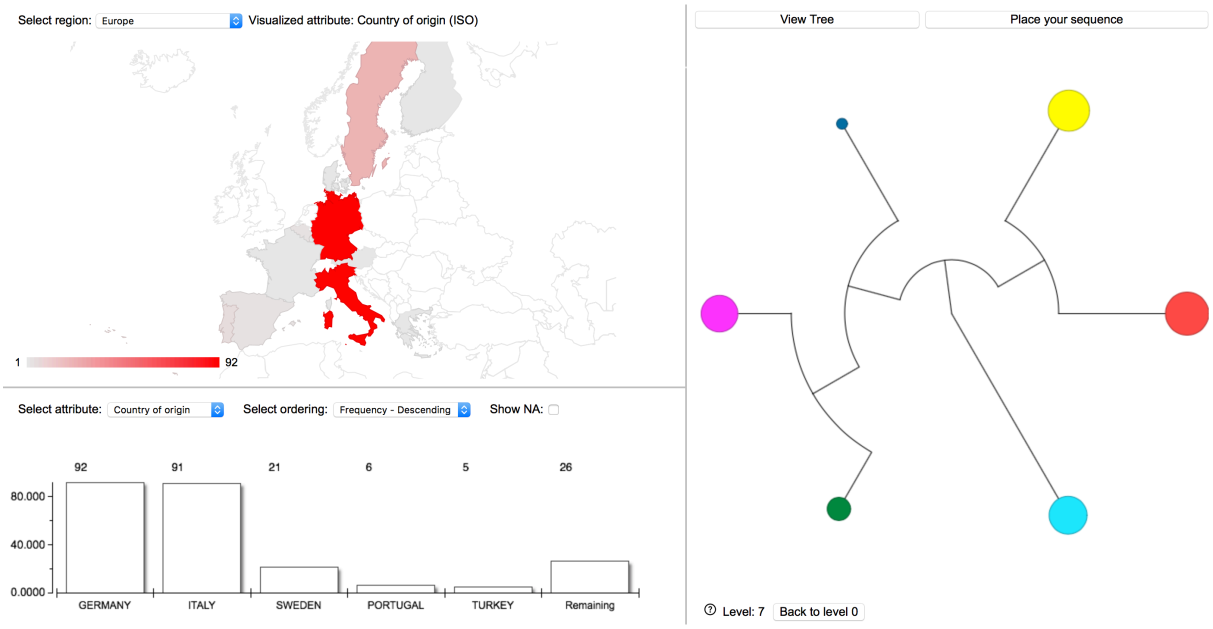

Figure S13. Frequency of country of origin and of TDR NRTI for the different clusters at level 7.

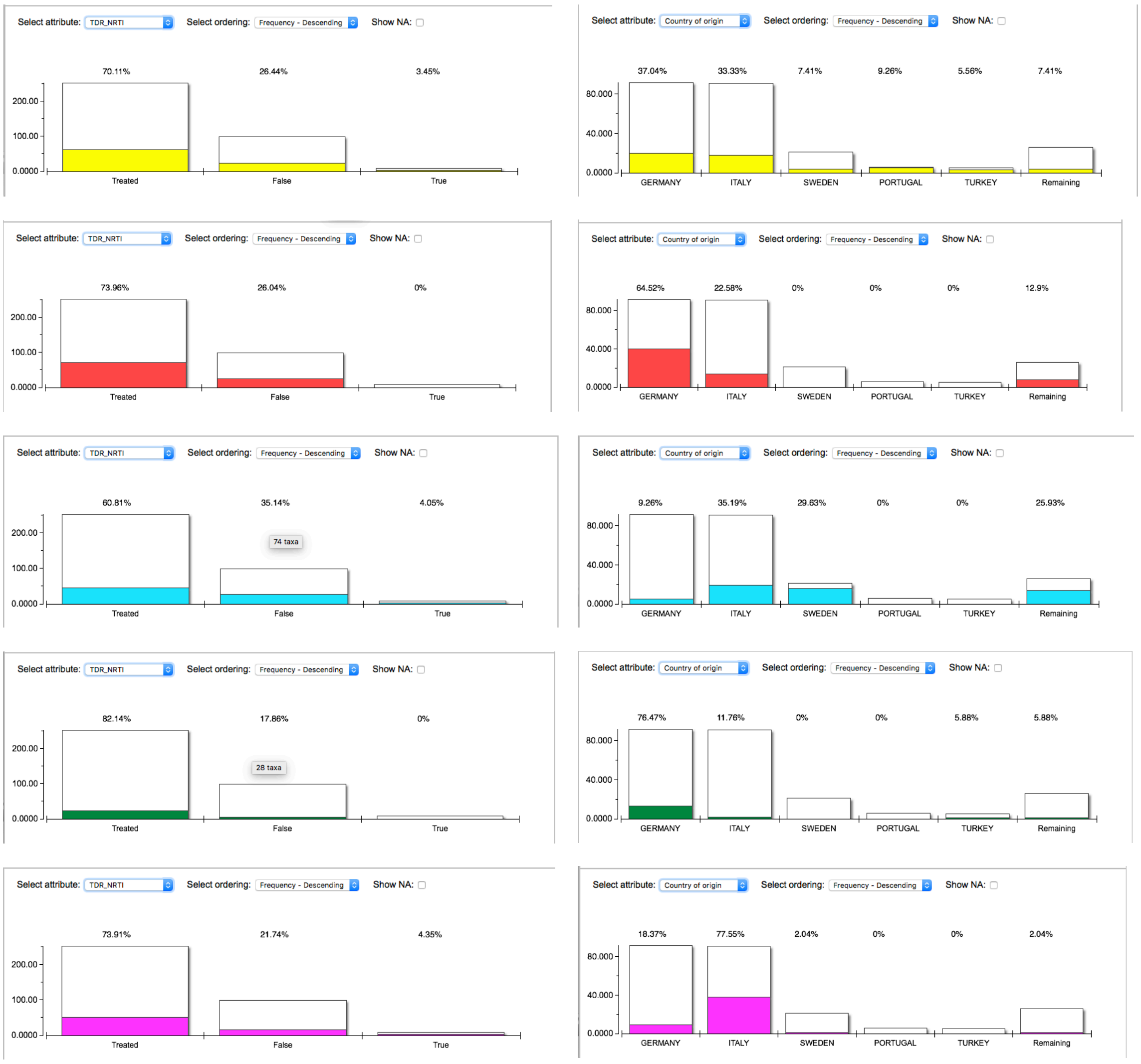

4. EucoHIV Study group

Chelsea & Westminster Hospital
London, United Kingdom
PI: Dr Mark Nelson
Study Group: Dr. Shin Yee Luk & Dr. Eleni Nastouli

Hospital Univesitario San Cecilio
Granada, Spain
PI: Dr. Federico Garcia
Study Group: Natalia Chueca, Marta Alvarez & Vicente Guillot

University of Nantes
Nantes, France
PI: Prof François Raffi
Study Group: Clotilde Allavena, Véronique Reliquet, Solène Pineau

IrsiCaixa Institute for AIDS Research
Hospital Universitari Germans Trias i Pujol
Badalona, Catalonia, Spain
PI: Dr Roger Paredes
Study group: Isabel Bravo and Rocío Bellido

North Middlesex University Hospital
London, UK
PI: Dr Jonathan Ainsworth
Study Group: Ms Anele Waters, Dr Achim Swenck

Guide Clinic, St. James's Hospital
Dublin, Ireland
PI: Dr. Fiona Mulcahy
Study group: Ms Siobhan O Dea

PZB Aachen
Aachen, Germany
PI: Dr Patrick Braun
Study group: Dr Heribert Knechten

Royal Liverpool University Hospital
Liverpool, United Kingdom
PI: Anna Maria Geretti
Study group: Ms Rachael Jones

Royal Free Hampstead NHS Foundation Trust
London, United Kingdom
PIs: Dr Daniel Webster and Prof Margaret Johnson
Study Group: Dr Ana Garcia

University Hospitals Leuven
Leuven, Belgium
PI: Prof Anne-Mieke Vandamme
Study Group: Dr Kristel Van Laethem

King's College Hospital
London, United Kingdom
PI: Dr Frank Post
Study Group: Dr Sudhanva Malur

Hospital de la Victoria
Malaga, Spain
PI: Dr Isabel Viciana
Study Group: Mrs Carmen González

Carlos III Hospital
Madrid, Spain
PI: Vicente Soriano
Study Group: Carmen de Mendoza, Rocio Sierra

Western General Hospital
Edinburgh, United Kingdom
PI: Prof Clifford Leen
Study Group: Ms Sheila Morris

St Mary's Hospital
Imperial Healthcare, London, UK
PI: Dr Nicola Mackie
Study Group: Drs Steve Keye, Jonathan Underwood, Borja Mora Peris, Jaime Vera, Killian Quinn

Luigi Sacco University of Milan, Italy
PI: Dr Stefano Rusconi
Study Group: Drs Paola Meraviglia, Valeria Micheli, Alessandro Mancon, Davide Mileto

Birmingham Heartlands Hospital, Birmingham, UK
PI: Dr Steve Taylor
Study Group: Dr Erasmus Smit, Justin Barnes

Institute of Infectious Diseases, University of Sassari
PI: Dr Giordano Madeddu

Ospedale Amedeo di Savoia, Torino, Italy
PI: Dr Letizia Marinaro
Study Group: Dr Stefano Bonora

Ghent University Hospital, Belgium
PI: Dr Linos Vandekerckhove
Study Group: Chris Verhofstede

Centre de Recherche Public de la Santé (CRP-Santé), Luxembourg
PI: Dr Carole Devaux

5. References

Rambaut, A., Robertson, et al. (2001) Phylogeny and the origin of HIV-1. Nature, 410, 1047-1048.

Matsen, F. A., et al. (2010). pplacer: linear time maximum-likelihood and Bayesian phylogenetic placement of sequences onto a fixed reference tree. BMC Bioinformatics, 11, 538.

Zazzi, M., et al. (2012) Predicting response to antiretroviral treatment by machine learning: the EuResist project. Intervirology, 55(2), 123-127.

Bennett, D. E., et al. "Recommendations for surveillance of transmitted HIV drug resistance in countries scaling up antiretroviral treatment." *Antiviral therapy* 13 (2008): 25.

Libin, P., et al. (2013). RegaDB: Community-driven data management and analysis for infectious diseases. *Bioinformatics*, *29*(11), 1477–1480.

Hofstra, L. Marije, et al. "Transmission of HIV drug resistance and the predicted effect on current first-line regimens in Europe." *Clinical infectious diseases* 62.5 (2016): 655-663.
